# Polyunsaturated fatty acids-induced ferroptosis suppresses pancreatic cancer growth

**DOI:** 10.1038/s41598-024-55050-4

**Published:** 2024-02-22

**Authors:** Akane Suda, Banlanjo Abdulaziz Umaru, Yui Yamamoto, Hiroki Shima, Yuriko Saiki, Yijun Pan, Liang Jin, Jiaqi Sun, Yi Ling Clare Low, Chitose Suzuki, Takaaki Abe, Kazuhiko Igarashi, Toru Furukawa, Yuji Owada, Yoshiteru Kagawa

**Affiliations:** 1 Florey Institute of Neuroscience and Mental Health, University of Melbourne, Parkville, VIC 3052 Australia; 2 Department of Organ Anatomy, Graduate School of Medicine, Tohoku University, Sendai, Miyagi 980-8575 Japan; 3 Center for Childhood Cancer Research, Nationwide Children’s Hospital, Columbus, OH 43205 USA; 4 Department of Biochemistry, Tohoku University Graduate School of Medicine, Sendai, 980-8575 Japan; 5 Department of Investigative Pathology, Tohoku University Graduate School of Medicine, Sendai, Miyagi 980-8575 Japan; 6 Drug Delivery Disposition and Dynamics, Monash Institute of Pharmaceutical Sciences, Monash University, Parkville, VIC 3052 Australia; 7 Department of Nephrology, Endocrinology and Vascular Medicine, Tohoku University Graduate School of Medicine, Sendai, 980-8575 Japan

**Keywords:** Pancreatic cancer, Drug resistance, Poly unsaturated fatty acids, Linoleic acid, α-Linolenic acid, Ferroptosis, Cancer, Cell biology, Molecular biology, Oncology

## Abstract

Despite recent advances in science and medical technology, pancreatic cancer remains associated with high mortality rates due to aggressive growth and no early clinical sign as well as the unique resistance to anti-cancer chemotherapy. Current numerous investigations have suggested that ferroptosis, which is a programed cell death driven by lipid oxidation, is an attractive therapeutic in different tumor types including pancreatic cancer. Here, we first demonstrated that linoleic acid (LA) and α-linolenic acid (αLA) induced cell death with necroptotic morphological change in MIA-Paca2 and Suit 2 cell lines. LA and αLA increased lipid peroxidation and phosphorylation of RIP3 and MLKL in pancreatic cancers, which were negated by ferroptosis inhibitor, ferrostatin-1, restoring back to BSA control levels. Similarly, intraperitoneal administration of LA and αLA suppresses the growth of subcutaneously transplanted Suit-2 cells and ameliorated the decreased survival rate of tumor bearing mice, while co-administration of ferrostatin-1 with LA and αLA negated the anti-cancer effect. We also demonstrated that LA and αLA partially showed ferroptotic effects on the gemcitabine-resistant-PK cells, although its effect was exerted late compared to treatment on normal-PK cells. In addition, the trial to validate the importance of double bonds in PUFAs in ferroptosis revealed that AA and EPA had a marked effect of ferroptosis on pancreatic cancer cells, but DHA showed mild suppression of cancer proliferation. Furthermore, treatment in other tumor cell lines revealed different sensitivity of PUFA-induced ferroptosis; e.g., EPA induced a ferroptotic effect on colorectal adenocarcinoma, but LA or αLA did not. Collectively, these data suggest that PUFAs can have a potential to exert an anti-cancer effect via ferroptosis in both normal and gemcitabine-resistant pancreatic cancer.

## Introduction

Pancreatic cancer often arises from microscopic non-invasive epithelial proliferations in the pancreatic ducts and are accompanied by driver gene mutations including *KRAS, TP53, SMAD4* and *CDKN2*^1^. With no early signs and poor clinical prognosis, pancreatic cancer rapidly spreads to surrounding organs, reaching an advanced stage before diagnosis and thus making surgical treatment challenging^2,3^. Despite advances in science and medical technology in recent years, only 20% of patients are eligible for resection, while 35% of patients are unresectable or locally advanced, and the rest are metastatic^4,5^. Therefore, adjuvant chemotherapy has become the selective treatment after surgery. However, gemcitabine chemotherapy which was implemented as standard of care in first-line treatment of metastatic pancreatic cancer, had a median overall survival of only 6.8 months^6,7^. Recently, the triple chemotherapy 5-FU, leucovorin, irinotecan and oxaliplatin (FOLFIRINOX), which can provide 11.1 months of median overall survival, is the actual treatment of choice for borderline resectable pancreatic cancers^8^. However, anticancer agents in chemotherapy can be a risk for acquiring drug-resistance in cancers and cause severe side effects including allergic reactions, hypotension, arrhythmia, dyspnea, nausea and vomiting. Therefore, an establishment of more effective treatment is urgently required.

Ferroptosis is a regulated form of necrotic cell death that exhibits unique morphological, biochemical and genetic features compared to previously characterized cell death^9^.Ferroptosis is mainly embodied by accumulation of iron and reactive oxygen species (ROS), leading to phospholipids oxidization. This chain reaction leads to the rupture of organelles and cell membrane resulting in cell death^10^. Under physiological conditions, ferroptosis is prevented by antioxidant enzymes including glutathione peroxidase 4 (GPX4), which has been reported as a master regulator in the ferroptosis process by interrupting lipid peroxidation and converting lipid hydroperoxide into non-toxic lipid alcohol^11^. In addition, ferroptosis suppressor protein (FSP1) has been shown to function as an oxidoreductase that reduces coenzyme Q10 (CoQ), which acts as a lipophilic radical-trapping antioxidant that suppresses lipid peroxides formation^12^. Recently, several studies have demonstrated the exploitation of these ferroptosis-resistance molecules as potential therapeutic targets for eradicating the carcinogenic cells^13^. In fact, the blockade of GPX4 suppressed the growth of gastric cancer^14^ and the blockade of FSP1 suppressed the growth of non-small cell lung cancer^15^ and hepatocellular carcinoma^16^. Furthermore, GPX4 inhibition can induce the drug sensitivity in tumors with anti-tumor drug resistance^17^, suggesting that ferroptosis inducer can develop an innovative tumor treatment. However, the major ferroptosis inducers experimentally used in in vitro studies do not yet meet pharmacokinetic standards for in vivo application due to poor water solubility and metabolic instability^18,19^. Therefore, it is highly desirable to identify effective molecules that can induce ferroptosis in vivo.

With respect to the number of double bonds present within the carbon chain, long chain fatty acids (LCFAs) are classified into saturated fatty acids (SFA) or unsaturated fatty acids. Unsaturated fatty acids can be further classified into monounsaturated fatty acids (MUFAs), including oleic acids (OA), and polyunsaturated fatty acids (PUFAs), including linoleic acid (LA) and α-linolenic acid (αLA). SFAs and MUFAs are the main components in cell and organelle membrane phospholipids and lipid droplet as well as suppliers of the energy source for β-oxidation in mitochondria^20,21^. PUFAs are also important fatty acids for membrane fluidity by being incorporated into membrane phospholipids^22^, although their amounts in phospholipids are significantly lower than SFA and MUFAs^23^. In addition, they serve as substrates for synthesis of specialized pro-resolving mediators that are important for homeostasis^24,25^. On the other hand, PUFAs are preferentially oxidized by lipid oxidation due to a reduction of bond strength accompanied by the increase in the degree of unsaturation or the number of double bonds^26^, suggesting that the structural stability of LCFAs might be highly related with the susceptibility of cells to ferroptosis. Recent studies have demonstrated that an increase in the synthesis of PUFAs promotes lipid peroxidation and produces ferroptosis death signals^27,28^, whereas the treatment of exogenous MUFAs inhibits ferroptosis by reducing PUFAs incorporation into phospholipids^29^, suggesting that PUFAs can have the therapeutic potential for tumor treatment. However, the underlying mechanisms and the specific species of PUFAs for anti-tumor effect are still unknown.

In this study, we explored the most effective FAs to suppress pancreatic cancer growth in vitro and the underlying mechanisms. In addition, using a pancreatic cancer xenograft model, we examined whether the anti-cancer effect of fatty acids treated through intraperitoneal injection can be preserved in vivo.

## Results

### LA and αLA pushed cell death into necroptotic/ferroptotic phenotypes

**Figure 1:**
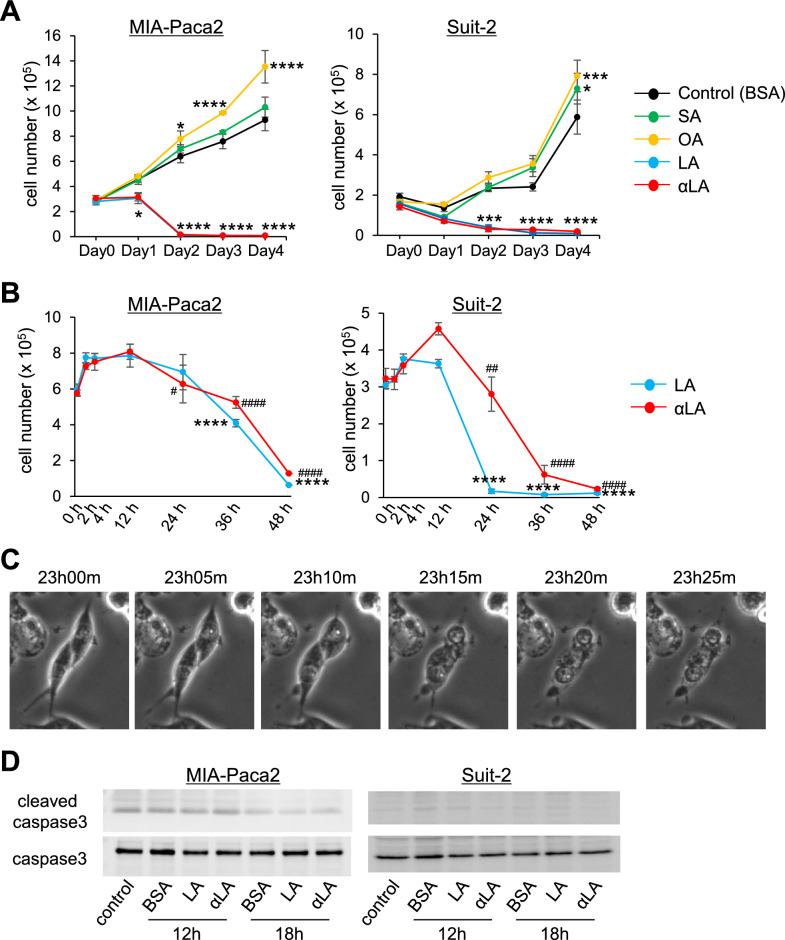
LA and αLA pushed cell death into necroptotic/ferroptotic phenotypes. (**A**) The proliferation rate of MIA-Paca2 and Suit-2 cells treated with BSA (control) or different 60 µM FAs (SA, OA, LA, and αLA). Live cells were counted successively for 4 days. One-way ANOVA followed by the Tukey test was used for multiple comparisons. Data shown are the means ± SEM *P < 0.05, ***P < 0.001, ****P < 0.0001 versus BSA-control. (**B**) The proliferation rate of MIA-Paca2 and Suit-2 cells treated with LA, and αLA. Live cells were counted in early time points up to 48 h. Data shown are the means ± SEM *P < 0.05, ****P < 0.0001 versus at 12 h in LA treatment, ^#^*P* < 0.05, ^##^*P* < 0.001, ^####^*P* < 0.00001 versus at 12 h in αLA treatment. (**C**) Representative phase contrast images showing the time course for the morphological change in Mia-Paca2 treated with LA. (**D**) Western blot for cleaved caspase 3 and caspase 3 in MIA-Paca2 and Suit-2 cells treated with BSA, LA, and αLA for 12 h and 18 h. Control is the cells treated with medium containing 1% FBS only.

To identify which FAs suppress pancreatic cancer cell growth, MIA-Paca2 and Suit-2 were treated with different 18-carbon FAs including SA, OA, LA and αLA for 4 days. Proliferation assay revealed a significant suppression of cell growth by LA and αLA treatment compared with BSA control at day 2, while from day 3, an entire cell death was observed in MIA-Paca2 cells (Fig. 1A). On the other hand, SA treatment did not affect the cell proliferation of MIA-Paca2, but the proliferation was significantly increased with OA treatment (Fig. 1A). Similarly, in Suit-2 cells, LA and αLA treatment induced cell death at day 2, while OA and SA significantly increased cell proliferation (Fig. 1A), suggesting that PUFAs possessing two or more double bonds can have the potentials to suppress the pancreatic cancer cell growth. Moreover, when we examined earlier time points for detailed changes below 48 h of treatment, we confirmed that the lethal event with LA and αLA occurred between 12 and 24 h after treatment (Fig. 1B). Timelapse imaging demonstrated that cells continued to proliferate until around 13 h, then started swelling, ballooning, and being ruptured (Fig. 1C, supplemental movie), which could be observed in the phase of necrosis^30^ as well as ferroptosis^31^. Furthermore, we did not observe any difference in cleaved caspase 3 levels between BSA- and LA/αLA-treated MIA-Paca2 or Suit2 cells (Fig. 1D), suggesting that apoptosis might not be in the cascade of LA and αLA- induced cell death.

### LA and αLA induced pancreatic cancer cell death through ferroptosis

**Figure 2:**
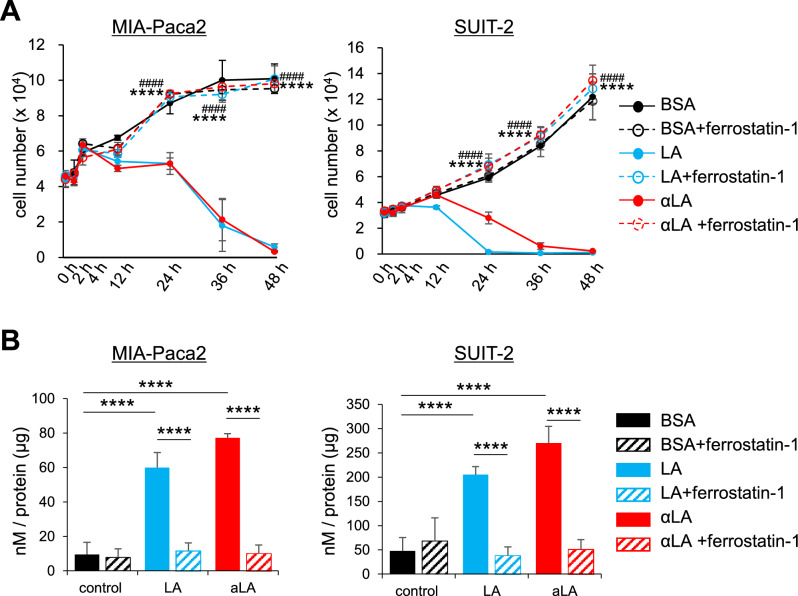
LA and αLA induced pancreatic cancer cell death through ferroptosis. (**A**) The proliferation rate of MIA-Paca2 and Suit-2 cells treated with BSA, 60 µM LA, and 60 µM αLA alone or together with 10 µM ferrostatin-1. Live cells were counted successively up to 48 h. One-way ANOVA followed by the Tukey test was used for multiple comparisons. Data shown are the means ± SEM ****P < 0.00001 versus with LA treatment without ferrostatin-1, ^####^*P* < 0.00001 versus with αLA treatment without ferrostatin-1 (**B**) Bar graph showing lipid peroxidation of LA and αLA in MIA-Paca2 and SUIT-2 cells treated with BSA, 60 µM LA, and 60 µM αLA alone or together with 10 µM ferrostatin-1. Data shown are the means ± SEM ****P < 0.00001.

Given that necroptotic/ferroptotic phenotypes were observed in LA and αLA treated pancreatic cancer cell lines, we investigated if ferrostatin-1, a ferroptosis inhibitor, could affect the pancreatic cancer cell growth in order to confirm the involvement of LA and αLA in ferroptosis pathway. Proliferation assay revealed that ferrostatin-1 did not change the proliferation rate of either MIA-Paca2 or Suit-2 when treated with BSA, however, the effect of LA and αLA on pancreatic cancer cell death was negated by treatment with ferrostatin-1, with proliferation rate being restored back to the same levels as BSA-treated control (Fig. 2A). Furthermore, upregulated lipid peroxidation by LA and αLA treatment in MIA-Paca2 or Suit-2 were also suppressed by ferrostatin-1 treatment to the same level with BSA control (Fig. 2B), suggesting that LA and αLA might induce pancreatic cancer cell death through ferroptosis.

### LA and αLA induced ferroptosis pathway leads to RIP/MLKL dependent necroptosis pathway

**Figure 3:**
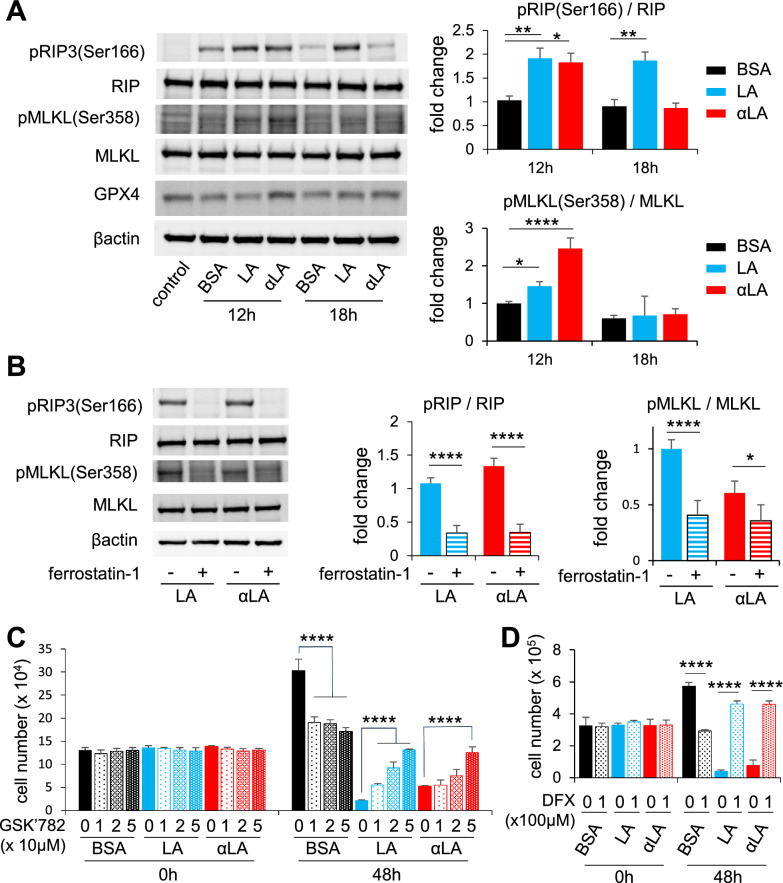
LA and αLA induced ferroptosis pathway leads to RIP/MLKL dependent necroptosis pathway. (**A**) Western blot for pRIP3 (Ser166), total RIP, pMLKL (Ser358), total MLKL, and GPX4 in MIA-Paca2 treated with BSA, 60 μM LA, and 60 μM αLA for 12 h and 18 h. Bar graphs show band density of relative phosphorylation levels analyzed using NIH-ImageJ. Data shown are the means ± SEM *P < 0.05, **P < 0.01, ****P < 0.0001 versus BSA-control. (**B**) Western blot for pRIP3 (Ser166), total RIP, pMLKL (Ser358) and total MLKL in MIA-Paca2 cells treated with 60 µM LA, and 60 µM αLA together with or without 10 µM ferrostatin-1 for 12 h. Bar graphs show band density of relative phosphorylation levels analyzed using NIH-ImageJ. Data shown are the means ± SEM *P < 0.05, ****P < 0.0001 versus BSA-control. (**C**) The proliferation rate of MIA-Paca2 and Suit-2 cells treated with BSA, 60 µM LA, and 60 µM αLA alone or together with GSK’782 (0, 10, 20, 50 µM). Live cells were counted at 0 h and 48 h. One-way ANOVA followed by the Tukey test was used for multiple comparisons. Data shown are the means ± SEM ****P < 0.00001 versus with LA or αLA treatment without GSK’782. (**D**) The proliferation rate of MIA-Paca2 and Suit-2 cells treated with BSA, 60 µM LA, and 60 µM αLA alone or together with 100 μM DFX. Live cells were counted at 0 h and 48 h. One-way ANOVA followed by the Tukey test was used for multiple comparisons. Data shown are the means ± SEM ****P < 0.00001 versus with LA or αLA treatment without DFX.

The molecular mechanism of necroptosis and ferroptosis has been under intensive investigation in recent years. The receptor-interacting protein (RIP) kinase, together with mixed lineage kinase domain-like pseudokinase (MLKL), are considered to be the key mediator of necroptosis^32,33^. Therefore, we assessed the phosphorylation of RIP3 and MLKL in MIA-Paca2 at 12 h and 18 h after FAs treatment by Western blot, and we observed increased phosphorylation levels of RIP3 at 12 h and 18 h after LA treatment and only at 12 h after αLA treatment (Fig. 3A). Similarly, the phosphorylation level of MLKL was significantly increased at 12 h after LA and αLA treatment (Fig. 3A). Furthermore, in consistence with our proliferation results, LA and αLA-induced upregulation of phosphorylation levels on RIP3 and MLKL were negated by treatment with ferrostatin-1 at 12 h (Fig. 3B). We also investigated if necrosis inhibitor GSK’782 could reverse the effect of LA and αLA-induced cell death. Proliferation assay revealed that GSK’782 partially reduced the rate of cell death induced by LA and αLA in a dose dependent manner, although it did not return to the levels observed in the BSA treated cells (Fig. 3C). In addition, although we could not observe a change in GPX4 expression in LA and αLA treated cells (Fig. 3A), deferasirox (DFX), an iron chelator leading to the reduction of lipid peroxidation, successfully negated the LA and αLA-induced cell death in MIA-Paca2 cells (Fig. 3D). These results suggest that the effects of LA/αLA are induced through both a ferroptosis dependent pathway upstream of RIP/MLKL, and a ferroptosis/necroptosis-dependent pathway via the RIP/MLKL pathway.

### Intraperitoneal administration of LA and αLA suppressed the growth of pancreatic cancer in the xenograft model

**Figure 4:**
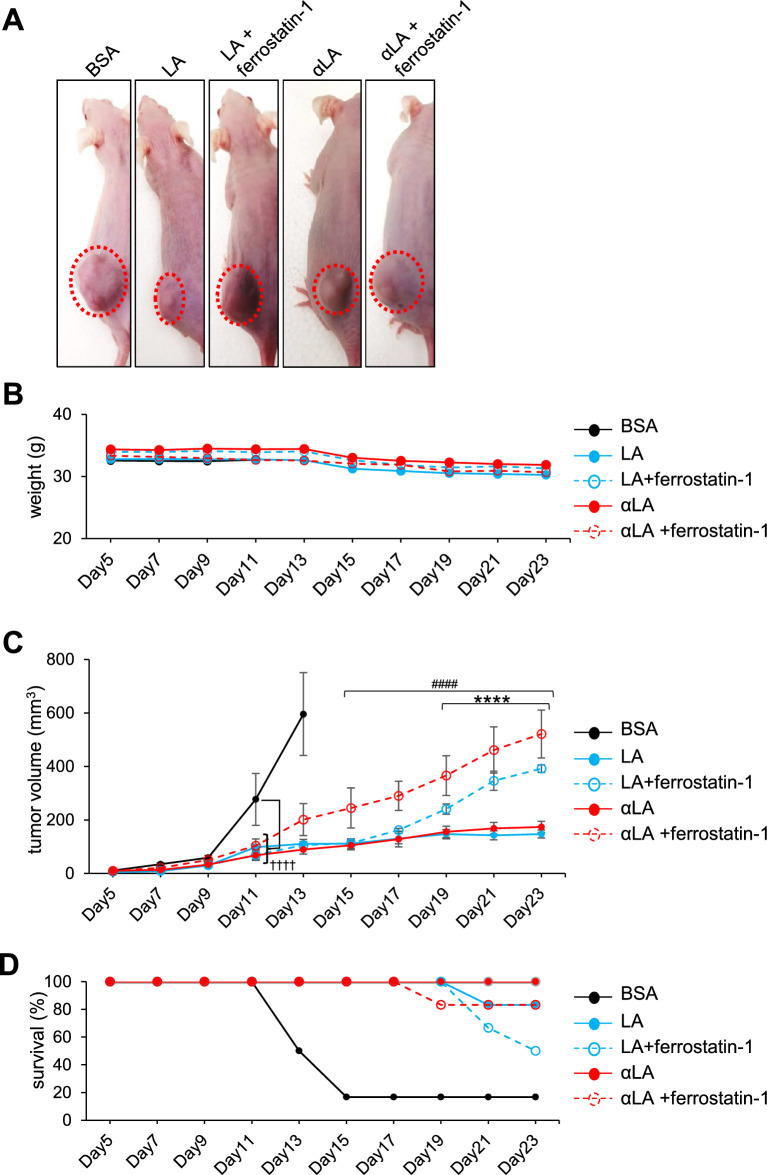
Intraperitoneal administration of LA and αLA suppressed the growth of pancreatic cancer in the xenograft model. (**A**) Representative images on tumor bearing mice treated with either BSA, 500 mg/kg LA, or 500 mg/kg αLA together with or without 20 mg/kg ferrostatin-1. (**B**) Average weight of mice treated with BSA as vehicle or 500 mg/kg LA, or 500 mg/kg αLA together with or without 20 mg/kg ferrostatin-1. (**C**) Cancer growth and (**D**) corresponding Kaplan–Meier survival curves in mice treated with either BSA, 500 mg/kg LA, or 500 mg/kg αLA together with or without 20 mg/kg ferrostatin-1. One-way ANOVA followed by the Tukey test was used for multiple comparisons. Data shown are the means ± SEM (n = 6 for each group) ****P < 0.00001 between with and without ferrostatin-1 in LA-treated group, ^####^*P* < 0.00001 between with and without ferrostatin-1 in αLA-treated group, ^┼┼┼┼^*P* < 0.00001 versus BSA-treated group.

Next, the roles of LA and αLA were examined in vivo using the Suit-2 subcutaneous xenograft model. In the mouse i.p. administrated with BSA, apparent cancer growth could be observed in 11 days after transplantation (Fig. 4A–C) and their survival rate started to be decreased at 13 days after transplantation (Fig. 4D). In contrast, i.p. injection of both LA and αLA significantly suppressed the cancer growth (Fig. 4A–C) and ameliorated the decreased survival rate of transplanted mice (Fig. 4D). Consistently with the in vitro results, ferrostatin-1 negated the anti-cancer effect of LA at 19 days after transplantation and the effect of αLA at 15 days after transplantation (Fig. 4A–C), resulting in decrease of survival rate (Fig. 4D).

### LA and αLA had different anti-cancer effects on Gemcitabine-resistant pancreatic cancers

**Figure 5:**
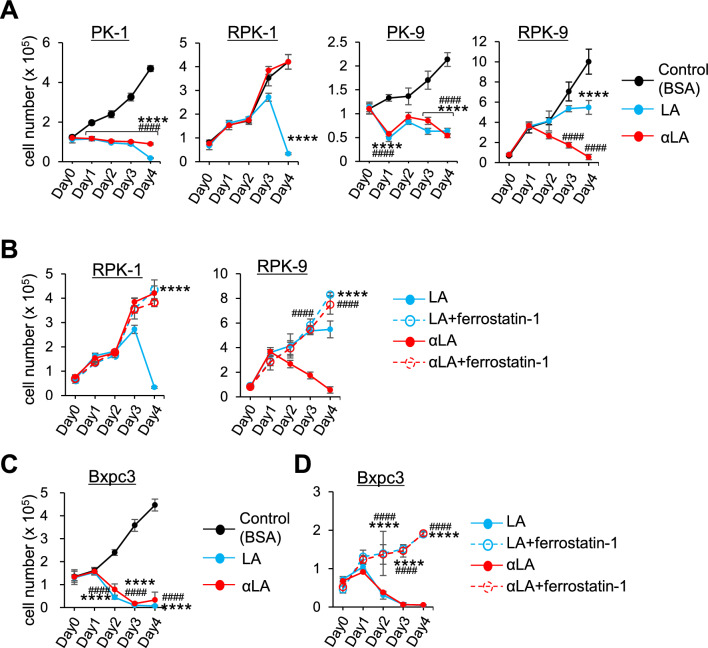
LA and αLA had different anti-cancer effects on Gemcitabine-resistant pancreatic cancers. (**A** and **C**) The proliferation rate of PK-1, RPK-1, PK-9 and RPK-9 cells (**A**) and BxPC3 cells (**C**) treated with BSA, 60 µM LA, and 60 µM αLA (**A**). Live cells were counted successively for 4 days. One-way ANOVA followed by the Tukey test was used for multiple comparisons. Data shown are the means ± SEM ****P < 0.00001 BSA-control versus LA, ^####^*P* < 0.00001 BSA-control and αLA. (**B** and **D**) The proliferation rate of RPK-1 and RPK-9 cells (**B**) and Bxpc3 cells (**D**) treated with 60 µM LA, and 60 µM αLA alone or together with 10 µM ferrostatin-1. Live cells were counted successively for 4 days. One-way ANOVA followed by the Tukey test was used for multiple comparisons. Data shown are the means ± SEM ****P < 0.00001 between with and without ferrostatin-1 in LA-treated cells, ^####^*P* < 0.00001 between with and without ferrostatin-1 in αLA-treated cells.

To examine the anti-cancer effect on drug-resistant pancreatic cancers, Gemcitabine-resistant cells RPK-1 and RPK-9, which we previously established^34^, were treated with LA and αLA. Additionally, given the predominance of KRAS mutation in pancreatic cancer cells, we used a Bcpx3 lacking the KRAS mutation to further evaluate the ferroptosis/necroptosis effect of LA and αLA. Proliferation assay revealed that LA and αLA successfully induced cell death in normal PK1 and PK9 cells similar to other pancreatic cancer cell lines including MIA-Paca2 and Suit-2, however the effect on RPK-1 and RPK9 was not consistent. Although LA induced cell death in RPK-1 4 days after treatment, αLA did not change the proliferation rate (Fig. 5A). On the other hand, in RPK-9, αLA induced cell death 3 days after treatment, but LA showed only a suppressive effect in cell growth compared with control (Fig. 5A). Interestingly, LA-induced cell death in RPK-1, suppression of proliferation in RPK-9, and αLA-induced cell death in RPK-9 were completely negated by ferrostatin-1, resulting in being restored back to the same levels as BSA-treated control (Fig. 5B). In the case of Bxpc3 cells, which do not possess *KRAS* mutation generally found in pancreatic cancers, LA and αLA induced an intensive effect of cell death via ferroptosis pathway (Fig. 5C,D). These results suggest that drug-resistant pancreatic cancers may have differential ferroptosis resistance with specific PUFA treatment.

### Ferroptosis effect by PUFA on pancreatic cancers is not dependent on the increasing number of carbon or double bonds in the PUFA

**Figure 6:**
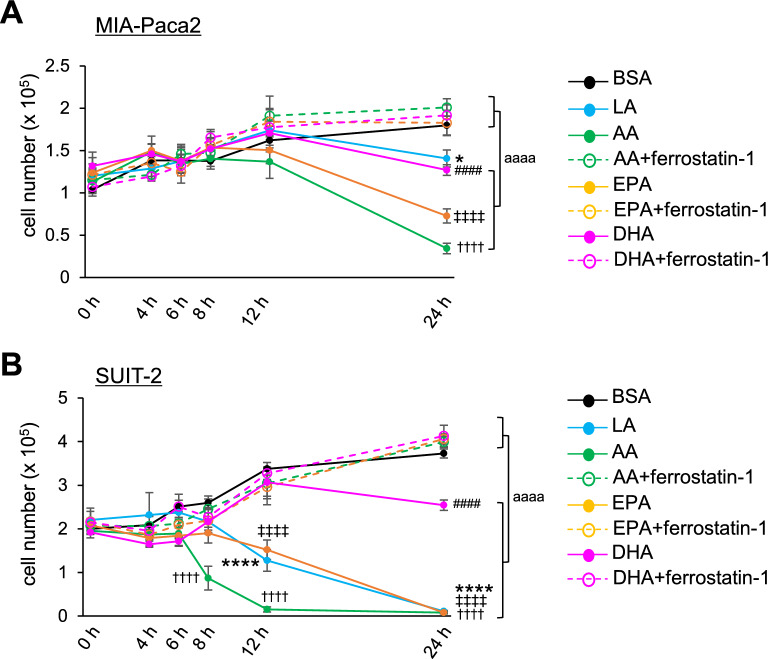
Ferroptosis effect by PUFA on pancreatic cancers is not dependent on the increasing number of carbon or double bonds in the PUFA. (**A** and **B**) The proliferation rate of MIA-Paca2 cells (**A**) and Suit-2 cells (**B**) treated with BSA, 60 µM LA, 60 µM AA, 60 µM EPA and 60 µM DHA alone or together with 10 µM ferrostatin-1. Live cells were counted successively up to 24 h. One-way ANOVA followed by the Tukey test was used for multiple comparisons. Data shown are the means ± SEM *P < 0.00001, ^####^*P* < 0.00001, ^┼┼┼┼^*P* < 0.00001, ǂ ǂ ǂ ǂ *P* < 0.00001 BSA-control versus LA, DHA, AA, EPA, respectively. ^aaaa^*P* < 0.00001 between with and without ferrostatin-1 treatment.

Since double bonds possessed by PUFAs are prone to oxidation, it is important to examine the correlation between the number of double bonds in PUFAs and susceptibility of pancreatic cancers to ferroptosis. Among the commonly known and researched PUFAs, arachidonic acid (AA, 20:4), eicosapentaenoic acid (EPA, 20:5) and docosahexaenoic acid (DHA, 22:6) have been shown by previous studies to have some anti-tumor properties^35^. Thus, we assessed the role of these PUFAs on pancreatic cancer cell growth. In MIA-Paca2, AA, EPA and DHA demonstrated an anti-cancer effect more clearly compared to LA (Fig. 6A). On the other hand, in Suit-2 cells, DHA treatment suppressed cell growth compared to BSA control without causing apparent cell death even at 24 h, while AA induced cell death already at 8 h after treatment (Fig. 6B). EPA treatment induced the cell death at the similar level as with LA treatment (Fig. 6B).

### Differential sensitivity to PUFA-induced ferroptosis in lung, skin, liver, colon and brain tumors

**Figure 7:**
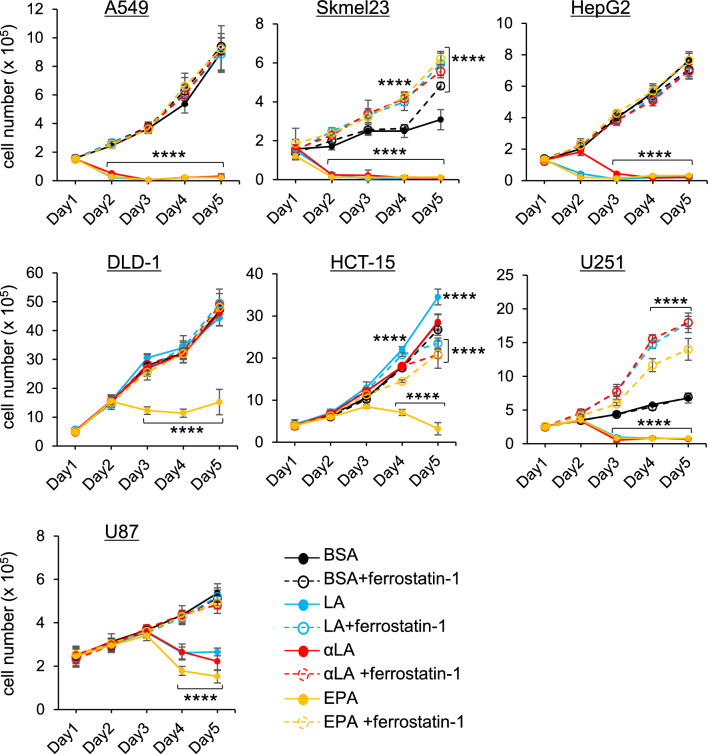
Different sensitivity of PUFA-induced ferroptosis. The proliferation rate of A549, Skmel23, HepG2, DLD-1, HCT-15, U251, and U87 cells treated with BSA, 60 µM LA, 60 µM AA, 60 µM αLA, and 60 µM EPA alone or together with 10 µM ferrostatin-1. Live cells were counted successively for 5 days. *P < 0.00001 versus BSA-control.

To understand the anti-tumor effect on other types of tumor cells including lung, skin, liver, colon and brain tumors, different cell lines were treated with LA, αLA and EPA. In A549 cells (human carcinoma), Skmel23 cells (human melanoma) and HepG2 cells (human hepatoma), all FAs exhibited marked effects on cell death (Fig. 7). On the other hand, LA and αLA were ineffective in the colon cancers DLD-1 or HCT15 (human colorectal adenocarcinoma), while EPA caused cell death to both cell lines (Fig. 7). In brain tumor cells, all the FAs showed cell death in U251 cells (human glioblastoma) at day 2 after treatment but could only partially suppress the tumor growth in U87 cells (human glioma) (Fig. 7).

## Discussion

Extensive studies have recently suggested that ferroptosis plays a pivotal role in tumor suppression and could be harnessed for cancer therapy^36,37^. Ferroptosis is defined as an intracellular iron-dependent form of cell death, and is distinct from other forms of cell death, including apoptosis and necrosis. However, in recent years, several studies have reported a crosstalk between necrosis and ferroptosis phenotypes. Although the activation of RIP1 and RIP3 kinase has been recognized as the key signaling in the necrosis pathway, Tong et al. demonstrated that RIP activation could be observed in RSL3 (a classic ferroptosis inducer)-induced ferroptosis^38^. In addition, GPX4 is proposed to be a master regulator in the ferroptosis process with a unique function to interrupt lipid peroxidation^11^ and GPX4-independent ferroptosis pathways, including enzymatic, metabolomic and lipidomic pathways, have been recently discovered in various physiological and pathophysiological processes^12,39–41^. Despite the numerous researches in recent years focusing on both ferroptosis and necrosis, the mechanism behind these cell death processes is only partially understood. However, what is importantly common between ferroptosis and necrosis is the increase in lipid peroxidation in the cell membrane, which triggers the cell swelling, ballooning, and rupture, as observed in Fig. 1C and supplemental movie.

**Figure 8:**
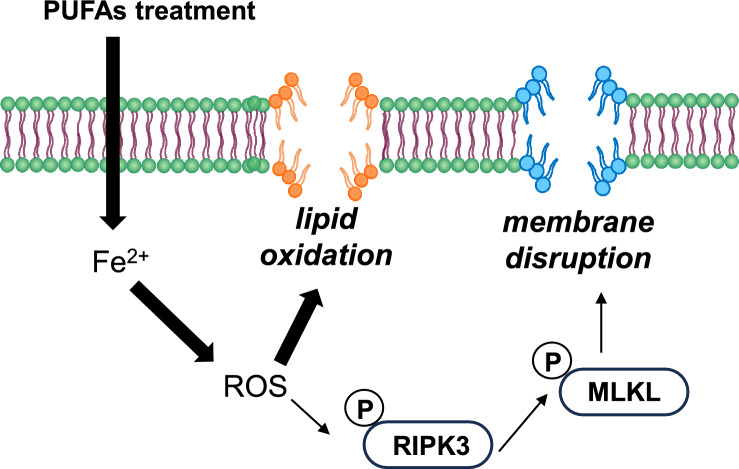
Schematic illustration how PUFAs induces ferroptotic cell death in the pancreatic cancers. Exogenous PUFAs including LA and αLA taken up into the cells undergo lipid peroxidation leading to ferroptotic cell death. In addition, ROS accumulation from LA and αLA peroxidation may induce phosphorylation of RIP3 and MLKL leading to membrane disruption.

So far, numerous studies over several decades have suggested potential anti-tumor effects of PUFAs using multiple targets, including transcriptional and mitochondrial regulation, anti-inflammation, including cell apoptosis as well as necroptosis^42,43^. Based on numerous recent researches, PUFAs are considered to be ultimate triggers of ferroptosis because increased lipid absorption can enrich the PUFA content of cell membranes^44^, driving the accumulation of lipid peroxidation and excess iron which produces ROS^10^. This ultimately leads to the porosity, destruction of the barrier function, and alteration in membranes permeability^45,46^. Here, we found that the treatment of LA and αLA among 18-carbon FAs increased lipid peroxidation (Fig. 2B) and suppressed pancreatic cancer growth with necroptotic/ferroptotic morphological change (Fig. 1A–C). Previous studies have shown that ferrostatin-1 is able to slow the accumulation of lipid hydroperoxides by acting as a radical-trapping antioxidants^47^ and also able to prevent the oxidative destruction of membrane lipid PUFAs^48^. In this study, ferrostatin-1 expectedly negated the increase of lipid peroxidation and cell death by LA and αLA treatment, restoring the levels back to the normal cell proliferation rate (Fig. 2A,B). Similarly to our results, molecular biological studies on ferroptosis in pancreatic cancers revealed that ARF6-silencing increased the expression of ACSL4, which enrich cellular lipid membrane with long PUFAs^27^, resulting in RLS3-induced ferroptotic cell death of PANC-1 and MIA-Paca2^49^. In addition, MGST1 limits lipid peroxidation via interaction with ALOX5, resulting in inhibition of ferroptotic pancreatic cancer cell death^50^. These results suggest that PUFAs enriched in pancreatic cancer cell membrane might induce ferroptosis/necrosis, possibly through excess ROS production derived from lipid peroxidation. We further demonstrated that ferrostatin-1 negated the LA and αLA-induced increased phosphorylation of both RIP3 (Ser166) and MLKL (Ser358), restoring back to normal cell proliferation rate in MIA-Paca2 (Fig. 3A,B). In addition, necroptosis inhibitor GSL’782 partially suppressed the LA and αLA-cell death although it did not return to the same level with BSA control (Fig. 3C). Collectively, these results indicate that LA/αLA induced lipid peroxidation is upstream of RIP/MLKL dependent necrosis pathway. Therefore, PUFA induced cell death might be dependent on both ferroptosis and necroptosis (Fig. 8).

Given that development of resistance to cancer therapy remains a major challenge, a number of preclinical and clinical studies have focused on overcoming drug resistance. Although gemcitabine is the main chemotherapy treatment for pancreatic cancer, resistance to gemcitabine begins to develop after a few weeks of treatment^51^. Gemcitabine, a deoxycytidine analog, has its main effects on DNA synthesis and promots DNA damage. However, long usage of gemcitabine leads to the accumulation of cytidine deaminase (CDA), which enhances drug detoxification of gemcitabine itself^52^. Other studies have demonstrated that excessive activation of PI3K/AKT and nuclear factor-κ-gene binding (NF-κB) signaling can lead to gemcitabine resistance in pancreatic cancers^53,54^. Although multiple mechanisms of gemcitabine resistance have been identified, we hypothesized that direct incorporation of PUFAs into gemcitabine-resistant pancreatic cancers can cause ferroptosis cell death similarly to other drug-sensitive pancreatic cancer cells including MIA-Paca2 and Suit-2. Unexpectedly, LA induced cell toxicity in RPK-1 and suppressed cell growth in RPK9 at 3–4 days after treatment, while αLA induced cell toxicity in RPK-9, but did not affect the RPK-1 growth (Fig. 5A). Ferrostatin-1 successfully restored these anti-cancer effect back to the control levels, suggesting that specific PUFAs partially induce ferroptosis cell death in gemcitabine-resistant pancreatic cancer cells. Considering the effect of gemcitabine in DNA synthesis, consequent altered lipid metabolism in RPK-9 may selectively affect the anti-cancer effect of αLA. In fact, Doll et al. have demonstrated that the ACSL4-deficient Pfa1 cells (TAM-inducible disruption of Gpx4 in mouse embryonic fibroblast cells) are refractory to ferroptosis, which can be re-sensitized by re-expression of ACSL4^27^. Other studies also demonstrated increased levels of fatty acid synthase (FASN) in gemcitabine-resistant pancreatic cancers, and FASN silencing downregulated the resistance^55^. These pieces of evidence together with our results may suggest that the difference in lipid metabolism between normal PK cells and gemcitabine-resistant PK cells exhibits different sensitization to ferroptosis induced by PUFAs including LA and αLA.

Our challenge to examine the correlation between the number of double bonds in PUFAs and the susceptibility of pancreatic cancers to ferroptosis demonstrated unexpected results. Considering the pro-inflammatory pathway of ROS production from AA, it was reasonable that AA exhibited a strong ferroptotic effect on MIA-Paca2 and Suit2 cells (Fig. 6A,B). EPA also demonstrated ferroptotic effects attaining entire cell death at 24 h and at a similar level as with LA and AA in Suit-2 cells (Fig. 6B). However, DHA, which possesses a larger number of double bonds used in this study, showed a mild anti-cancer effect compared to the drastic effects of LA, AA and EPA (Fig. 6B). It should be noted that stressed cells caused by PUFA overload from the microenvironment activate protective membrane remodeling mechanisms to redistribute these PUFAs phospholipid membrane pools, and sequestered them into lipid droplets (LD) and TAGs pools, thereby preventing PUFA oxidation and cellular damage^56–59^. Moreover, it has been shown that the increase in LD formation in cells treated with FAs correlates with the number of double bonds in the FAs, and especially DHA-treated colorectal cancer cells (SiHa and HCT-116) showed the largest number of LDs formed^60^. Additionally, PUFAs are hydrolyzed by the phospholipase A2 (PLA_2_) enzymes and released from membrane phospholipids and then imported into cells through various fatty acid transport proteins, such as fatty acid translocase (FAT/CD36), fatty acid transport protein, and fatty acid binding protein^61,62^. Particularly, AA and EPA are preferentially released by cytoplasmic PLA_2_, whereas DHA is released by Ca^2+^-independent PLA_2_^63^. Together, these data corroborate our findings of DHA showing a mild effect on ferroptosis compared to other PUFAs. The mechanisms of how altered lipid metabolism differentiate the preference of FAs for ferroptosis will be further explored in future studies.

Increasing evidence has demonstrated that PUFAs play significant roles in tumor risk and progression^64^. However, despite numerous pre-clinical mechanistic studies using cell lines and mouse models to determine molecular targets of PUFAs, they have not been translated into clinical trials. Lipid metabolic alterations frequently occur in malignant tumors, and thus, understanding the mechanisms that maintain lipid homeostasis in drug-resistant tumor cells may reveal the metabolic characteristics that could be applied to the clinical setting. Having shown the effect of PUFAs in pancreatic cancer cells, we further challenged different tumor cell lines with PUFAs treatment (Fig. 7). We indeed observed a differential sensitivity to PUFA-induced ferroptosis in different cancer cell lines and these findings support the previous observation of PUFAs-induced ferroptotic cell death in tumors. In fact, docosapentaenoate (DPA, n-6 FA) and DHA treatment induced ferroptotic cell death in effect in HCT-116 cells (human colorectal carcinoma)^60^. Furthermore, in melanoma A375 and B16F10, AA synergizes with interferon (IFN)γ to induces ferroptotic cell death^65^. These results suggest that different tumors may have a specific preference in PUFAs uptake into cell membrane or may have differential effects on lipid metabolism. In this study, we demonstrated that only EPA induced ferroptosis in human colorectal carcinoma including DLD-1 and HCT-15, but not LA and αLA (Fig. 7). Moreover, previous studies have shown unique inherent susceptibility of different cancer cells to ferroptosis due to the differences in metabolic reprograming, imbalance in ferroptosis defenses and genetic mutations^66–68^. For example, it has been shown that in gastrointestinal cancer cells, DNA methylation-mediated silencing of very long-chain fatty acid protein 5 (ELOVL5) and fatty acid desaturase 1 (FADS1), as well as the inability of these cells to generate AA and adrenic acid (AdA) from LA to be oxidized by lipoxygenases, render them resistant to ferroptosis^66^. On the other hand, it has been shown that in isocitrate dehydrogenase 1 (IDH1) mutant cells such as glioblastomas, the oncometabolite, 2-Hydroxyglutarate, increases erastin-induced lipid ROS accumulation and sensitizes cells to ferroptosis^68^. As such, further research to elucidate the cancer type-specific requirement of different PUFAs for ferroptotic as well as necroptotic cell death is needed to advance therapeutic targeting of these diseases.

## Conclusion

In this study, we newly demonstrated that LA and αLA induced ferroptotic cell death in MIA-Paca2 and Suit-2, which is negated by ferrostatin-1 restoring back to BSA control levels. Consistently, intraperitoneally administration of LA and αLA suppressed subcutaneously transplanted Suit-2 cell growth in xenograft model mice. In addition, LA and αLA partially induced a ferroptotic effect on the gemcitabine-resistant-PK cells, although the effect was exerted late compared to treatment of normal-PK cells. The trial to validate the importance of double bonds in PUFAs in ferroptosis revealed that AA and EPA showed marked effects of ferroptosis on pancreatic cancer cells, while DHA showed mild suppression of cancer growth. Further challenges into other tumor types revealed different sensitivity of PUFA-induced ferroptosis. Taken together, these data suggest that PUFAs can have a potential to exert anti-cancer effect via ferroptosis in both normal and gemcitabine-resistant pancreatic cancers.

## Materials and methods

### Ethics statement

We confirmed that all methods were performed in accordance with the relevant guidelines and regulations. The research was carried out in compliance with the ARRIVE guidelines (https://arriveguidelines.org). This study also complies with all relevant ethical regulations in Japan. The animal protocols and procedures for experiments were approved by the Animal Committee of the Tohoku University School of Medicine Ethics Committee (2022MdA-012), and all experiments were conducted according to the ethical and safety guidelines of the Institute.

### Antibodies and reagents

Primary antibodies used in this study were rabbit polyclonal anti-cleaved caspase-3 (Asp175) (Cell Signaling Technology Inc, Beverly, MA, USA. Cat. No. 9661), rabbit polyclonal anti-caspase-3 (Cell Signaling Technology Inc. Cat. No. 9662), rabbit monoclonal anti-phospho-RIP3 (Ser227) (Cell Signaling Technology Inc. Cat. No. 93654), rabbit monoclonal anti-RIP (Cell Signaling Technology Inc. Cat. No. 73271), rabbit monoclonal anti-phospho-MLKL (Ser358) (Abcam, Cambridge, England, Cat. No. ab187091), rabbit monoclonal anti-MLKL (Abcam, Cat. No. ab184718), rabbit polyclonal anti-GPX4 (Cell Signaling Technology Inc. Cat. No. 52455) and mouse monoclonal anti-β actin (Santa Cruz, TX, USA, Cat. No. sc-47778). Secondary antibodies used in this study were goat anti-rabbit IgG (H + L) HRP conjugated (Merck Millipore, MA, USA, Cat. No. AP307P) and goat anti-mouse IgG (H + L) HRP conjugated (Merck Millipore, Cat. No. AP308P). Reagents used in this study were Ferrostatin-1 (Cayman chemical, MI, USA, Cat. No. 17729), GSK’782 (Selleck Chemicals, Pittsburgh, PA, USA. Cat. No. S8642), fatty acid-free Bovine serum albumin (Nacalai Tesque, Kyoto, Japan, Cat. No. 08587), stearic acid (Cayman Chemical, MI, USA, Cat. No. 10011298), oleic acid (Cayman Chemical, Cat. No. 90260), linoleic acid (Cayman Chemical, Cat. No. 90150), α-linolenic acid (Cayman Chemical, Cat. No. 90210), arachidonic acid (Cayman Chemical, Cat. No. 90010), eicosapentaenoic acid (Cayman Chemical, Cat. No. 90110) and docosahexaenoic acid (Cayman Chemical, Cat. No. 90310). DFX was provided as a raw material by Novartis Pharma.

### Cell culture and treatment

Human pancreatic cell lines MIA Paca2 and Suit-2, human lung cancer cell line A549, human hepatocellular carcinoma HepG2, human colon adenocarcinoma DLD-1, HCT-15, and Colo-205, and human glioblastoma cell line U87 were obtained from Cell Resource Centre for Biomedical Research in Tohoku University. Human melanoma cell lines SKmel23 were kindly provided by Prof. Yutaka Kawakami in Keio University^69^. Human glioblastoma cell line U251 was purchased from the JCRB Cell Bank (Osaka, Japan). Human pancreatic cancer cell lines and acquired gemcitabine-resistant human pancreatic cancer cell lines were established as described^34,70,71^. MIA Paca2, Suit-2, PK-1, RPK-1, PK-9, RPK-9 and Skmel23 were maintained in RPMI1640 Medium (Sigma Aldrich, Co., St. Louise, MO, USA) and other cell lines in Dulbecco’s modified Eagle’s medium (Sigma Aldrich, Co.) both supplemented with 10% (v/v) heat-inactivated fetal bovine serum (FBS) (Thermo Fisher Scientific Inc.) and 1% (v/v) penicillin/streptomycin (Thermo Fisher Scientific Inc.) at 37 °C with 5% CO_2_. All cell lines were routinely tested for mycoplasma by Polymerase chain reaction (PCR).

Cells were cultured in appropriate dishes and serum-deprived for 24 h prior to fatty acids (FAs) treatment. FAs were prepared in FA-free BSA-DPBS vehicle as described previously^59^. In brief, 1.2 mM FA stock was mixed with 3 mM FA-free BSA-DPBS vehicle and rotated for 1 h at 37 °C for conjugation. The FA mixture was then diluted in 1% FBS medium to a final concentration of 60 µM FA/ 150 µM BSA. 1% FBS medium containing 150 µM BSA was used as control. When required, ferrostatin-1 (10 μM), GSK’782 (10 nM, 20 nM, 50 nM) and DFX (100 μM) were co-treated with FA treatment.

### Cell proliferation assay

Cells were seeded in 12‐well plates at a density of 1 × 10^4^ cells/well. Cell proliferation was evaluated using an automated cell counter (CellDrop FL, DeNovix, DE, USA). Cells were counted using the Trypan blue exclusion staining to discriminate between live and dead cells. Cells were also washed twice to eliminate any dead cells before counting.

### Timelapse imaging

Cells were seeded in a 35-mm glass bottom dish at a density of 5 × 10^3^ cells/well. Twenty-four hours after serum deprivation, the dishes were placed in the microscope (BZ-X800, Keyence, Osaka, Japan) with a time-lapse imaging system. The cell tracking was started immediately after replacing the cultured medium with FA medium over 48 h.

### Lipid peroxidation assay

The levels of lipid peroxidation were evaluated using Lipid Peroxidation (MDA) Assay Kit (Abcam, Cat. No. ab118970) following manufactured protocols. The levels of lipid peroxidation were normalized by protein levels evaluated by BCA assay (Thermo Fisher Scientific Inc.) with BSA standards.

### Western blot

Western blot experiments were performed as previously described^72^. In brief, after preparing whole cell lysates using sodium dodecyl sulfate polyacrylamide gel electrophoresis (SDS-PAGE) sample buffer containing protease and phosphatase inhibitors (Roche, Basel, Switzerland), protein concentrations were measured by the BCA assay (Thermo Fisher Scientific Inc.) with BSA standards. Next, the lysates were resolved by SDS-PAGE and transferred to a polyvinylidene difluoride membranes (Merck Millipore). After blocking non-specific binding with 0.1% (v/v) Tween 20 containing 5% (w/v) bovine serum albumin in TBS for 1 h, the membranes were immunoblotted with primary antibodies overnight at 4 °C. The membranes were then incubated with HRP-conjugated secondary antibody for 2 h. Detection was performed with the ECL Western Blot Detection Kit (Thermo Fisher Scientific Inc.) using a Chemi-DOC system (Bio-Rad, Hercules, CA).

### Xenograft model

Adult male Crlj:SHO*-Prkdc*^*scid*^*Hr*^*hr*^ (Hairless SCID) (6 weeks) were obtained from Charles River Laboratories Japan, Inc. Surgical procedures were performed under sterile conditions after acclimatization for 2 weeks. In brief, Suit-2 cell lines (total 3.0 × 10^6^ cells; 200 µl of 1.5 × 10^7^ cells/ml in PBS) were subcutaneously injected into the left pelvic limb with a 25 G needle syringe under isoflurane inhalation anaesthesia. 5 days after surgery, mice were treated by intraperitoneal injection with 500 µl FA (500 mg/kg wight) conjugated with BSA in PBS, with or without 20 mg/kg ferrostatin-1 in 50% PEG300, 2% DMSO, 5% Tween80 and 43% water^60^. The treatment was done every other day until 23 days after surgery. The tumor volume was measured with a calliper and calculated according to the formula: V = (length [mm] × width [mm]^2^)/2. Mice were excluded from the experiment if they developed limp palsy, lost significant weight, or were unable to drink water.

### Statistical analysis

All data represent the mean ± s.e.m of at least three independent experiments. Statistical comparisons of means were made by Student’s two-tailed unpaired t-test or one-way ANOVA followed by the Tukey test for multiple comparisons. *P* values less than 0.05 were considered statistically significant.

### Supplementary Information

Supplementary Figures.

Supplementary Video 1.

Supplementary Legends.

## Data Availability

All data that support the findings of this study are available from the corresponding author on reasonable request.

## Abbreviations

AA: Arachidonic acid
αLA: α-Linolenic acid
BSA: Bovine serum albumin
DFX: Deferasirox
DHA: Docosahexaenoic acid
EPA: Eicosapentaenoic acid
FA: Fatty acid
FBS: Fetal bovine serum
FSP1: Ferroptosis suppressor protein 1
GPX4: Glutathione peroxidase 4
LA: Linoleic acid
LCFA: Long chain fatty acid
MLKL: Mixed lineage kinase domain-like pseudokinase
MUFA: Monounsaturated fatty acid
OA: Oleic acid
PUFA: Polyunsaturated fatty acid
RIP: Receptor-interacting protein
ROS: Reactive oxygen species
SA: Stearic acid
SFA: Saturated fatty acid
