# Supplementary material for: Polyunsaturated fatty acids-induced ferroptosis suppresses pancreatic cancer growth
Source: Sci Rep. 2024 Feb 22;14:4409. doi: 10.1038/s41598-024-55050-4 (PMC10884029; doi:10.1038/s41598-024-55050-4)
Supplement: Supplementary file 1 — Supplementary Figures. [file 41598_2024_55050_MOESM1_ESM.pdf]

Supplemental Fig.1

WB image (Fig. 1D) for cleaved caspase3 in MIA-Paca2 was cropped from this image

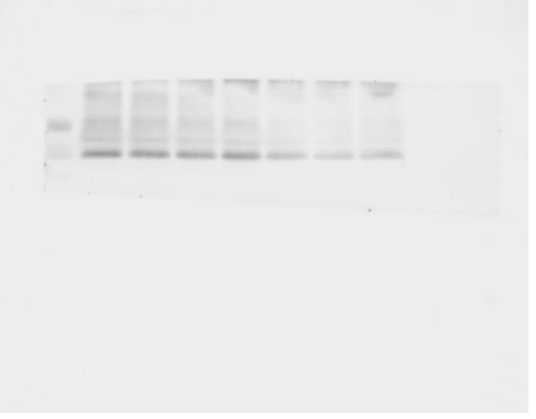

WB image (Fig. 1D) for cleaved caspase3 in Suit-2 was cropped from this image

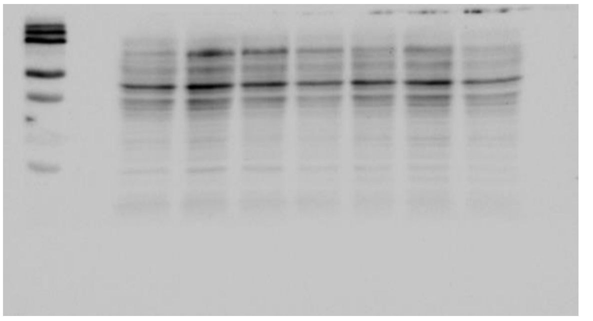

WB image (Fig. 1D) for caspase3 in MIA-Paca2 was cropped from this image

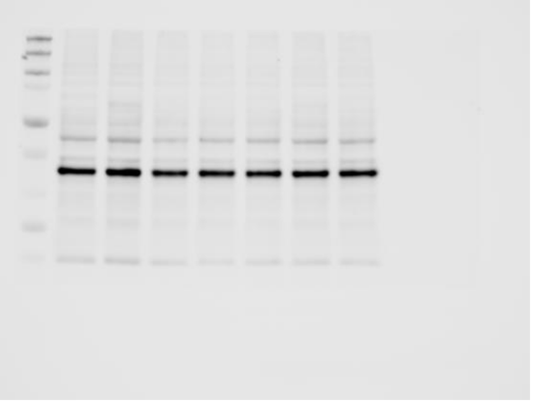

WB image (Fig. 1D) for caspase3 in Suit-2 was cropped from this image

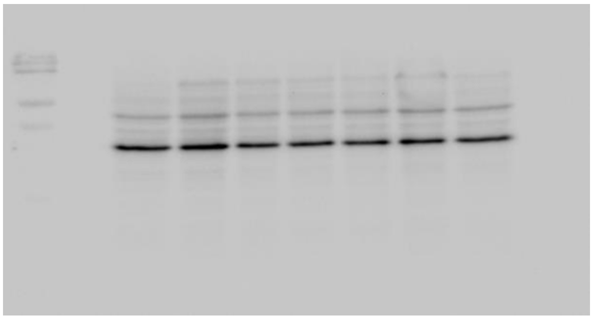

Supplemental Fig.2

WB image (Fig. 3A) for pRIP3 (Ser166)
was cropped from this image

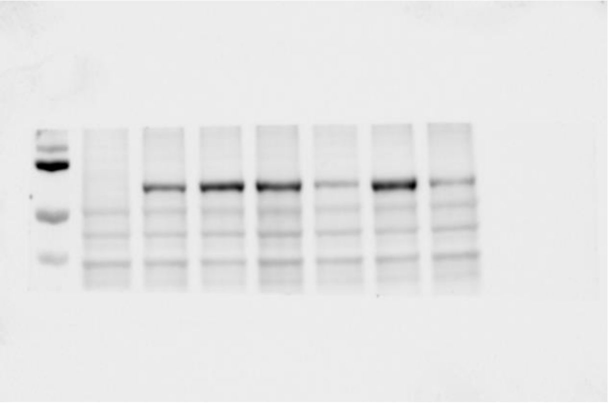

WB image (Fig. 3A) for RIP
was cropped from this image

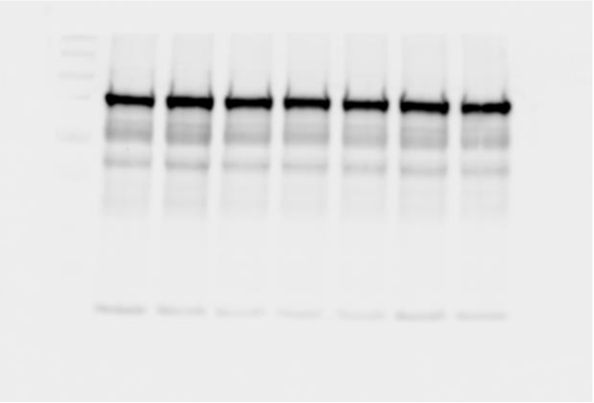

WB image (Fig. 3A) for pMLKL(Ser358)
was cropped from this image

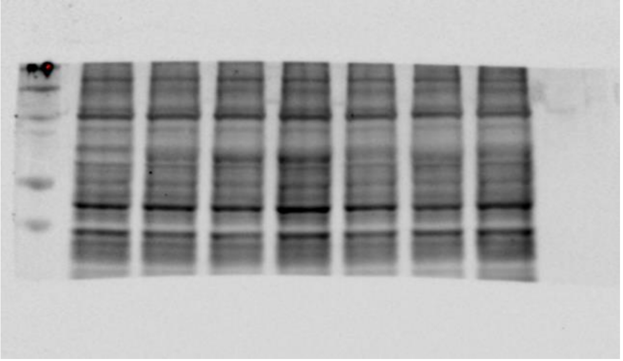

WB image (Fig. 3A) for MLKL
was cropped from this image

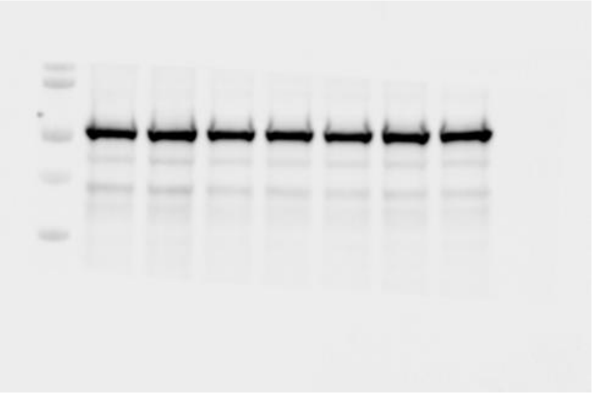

WB image (Fig. 3A) for GPX4
was cropped from this image

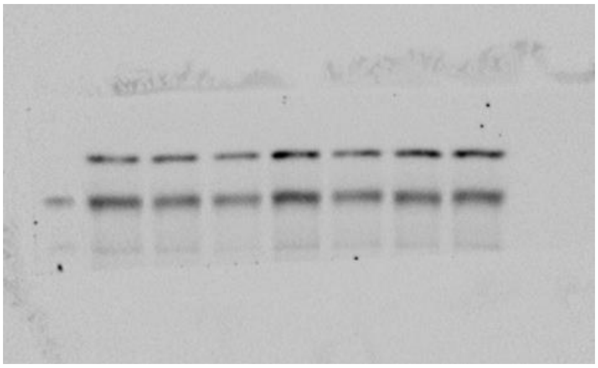

WB image (Fig. 3A) for βactin
was cropped from this image

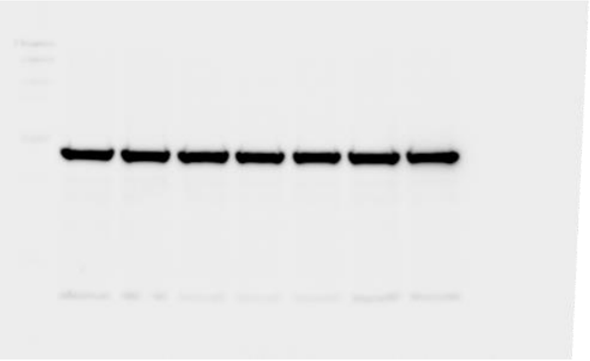

Supplemental Fig.2 continued

WB image (Fig. 3B) for pRIP3 (Ser166)
was cropped from this image

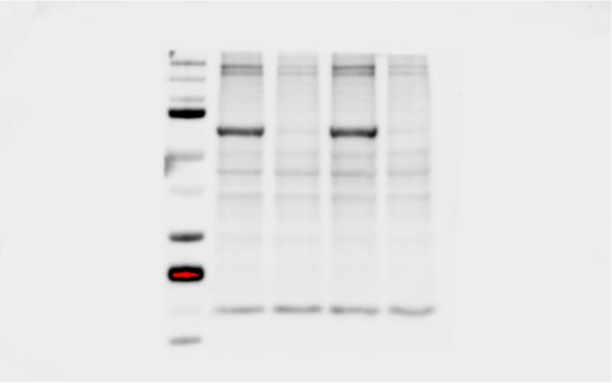

WB image (Fig. 3B) for RIP
was cropped from this image

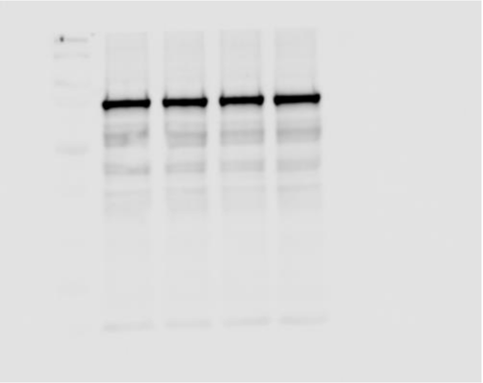

WB image (Fig. 3B) for pMLKL(Ser358)
was cropped from this image

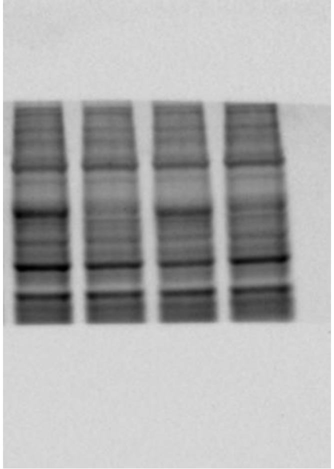

WB image (Fig. 3B) for MLKL
was cropped from this image

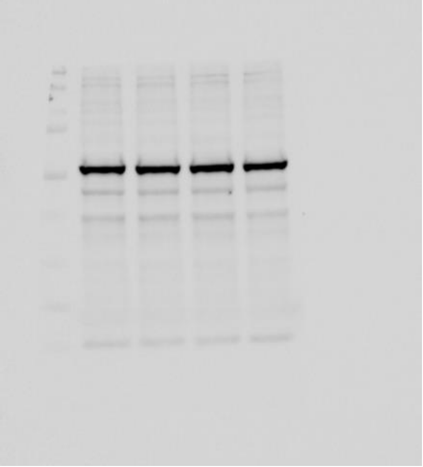

WB image (Fig. 3B) for βactin
was cropped from this image
