# Supplementary material for: Polyunsaturated fatty acids-induced ferroptosis suppresses pancreatic cancer growth
Source: Sci Rep. 2024 Feb 22;14:4409. doi: 10.1038/s41598-024-55050-4 (PMC10884029; doi:10.1038/s41598-024-55050-4)
Supplement: Supplementary file 3 — Supplementary Legends. [file 41598_2024_55050_MOESM3_ESM.pdf]

Supplementary Video Legend

Timelapse imaging of MIA-Paca2 cells treated with LA

Timelapse imaging were performed using the microscope (BZ-X800, Keyence, Osaka, Japan) with a time-lapse imaging system. The cell tracking was started immediately after replacing the cultured medium with FA medium over 48 hours. MIA-Paca2 cells treated with 60 µM LA continued to proliferate until around 13 h, then started swelling, ballooning, and being ruptured.
